# Effects of altitude on human oral microbes

**DOI:** 10.1186/s13568-021-01200-0

**Published:** 2021-03-06

**Authors:** Fang Liu, Tian Liang, Zhiying Zhang, Lijun Liu, Jing Li, Wenxue Dong, Han Zhang, Su Bai, Lifeng Ma, Longli Kang

**Affiliations:** 1 Key Laboratory for Molecular Genetic Mechanisms and Intervention Research on High Altitude Disease of Tibet Autonomous Region, School of Medicine, Xizang Minzu University, Xianyang, 712082 China; 2 Key Laboratory of High Altitude Environment and Genes Related to Diseases of Tibet Autonomous Region, School of Medicine, Xizang Minzu University, Xianyang, 712082 China; 3 Department of Pathology, The Second Affiliated Hospital of Shaanxi University of Chinese Medicine, Xianyang, 712000 China

**Keywords:** Oral microbiota, Altitude, Alpha diversity, Beta diversity, Network

## Abstract

Human oral microbes play a vital role maintaining host metabolic homeostasis. The Qinghai-Tibet Plateau is mainly characterized by a high altitude, dry, cold, and hypoxic environment. The oral microbiota is subject to selective pressure from the plateau environment, which affects oral health. Only a few studies have focused on the characteristics of oral microbiota in high-altitude humans. We collected saliva samples from 167 Tibetans at four altitudes (2800 to 4500 m) in Tibet to explore the relationship between the high altitude environment and oral microbiota. We conducted a two (high- and ultra-high-altitude) group analysis based on altitude, and adopted the 16S rRNA strategy for high-throughput sequencing. The results show that the alpha diversity of the oral microbiota decreased with altitude, whereas beta diversity increased with altitude. A LEfSe analysis revealed that the oral microbial biomarker of the high-altitude group (< 3650 m) was *Streptococcus*, and the biomarker of the ultra-high-altitude group (> 4000 m) was *Prevotella*. The relative abundance of *Prevotella* increased with altitude, whereas the relative abundance of *Streptococcus* decreased with altitude. A network analysis showed that the microbial network structure was more compact and complex, and the interaction between the bacterial genera was more intense in the high altitude group. Gene function prediction results showed that the amino acid and vitamin metabolic pathways were upregulated in the ultra-high-altitude group. These result show that altitude is an important factor affecting the diversity and community structure of the human oral microbiota.

**Supplementary Information:**

The online version contains supplementary material available at 10.1186/s13568-021-01200-0.

## Introduction

The oral cavity is exposed to the external environment and is an important organ connecting the external environment and the human respiratory and digestive systems (Diamond et al. 2008; Aleti et al. 2019). The oral cavity is the second largest microbial community, colonized with more than 700 different microbial species (Paster et al. 2006; Human Microbiome Project 2012). Oral microorganisms play an essential role maintaining oral homeostasis and preventing oral diseases (Gao et al. 2018). Moreover, the oral microbiota is influenced by hypoxia, low-temperature, host sex, age, and oral diseases (Grant et al. 2010; Lamont et al. 2018). Disorders of the oral microbiota are associated with various diseases, such as dental caries, periodontitis, peri-implantitis, mucosal diseases, and oral cancers (Jorth et al. 2014; Gao et al. 2018; Wasfi et al. 2018). An imbalance in the oral microbiota has been associated with metabolic diseases, such as inflammatory bowel disease, pancreatic cancer, diabetes, obesity, and cardiovascular disease (Fardini et al. 2010; Koren et al. 2010; Ahn et al. 2012; Jorth et al. 2014; Atarashi 2017; Peters et al. 2017; Lira-Junior and Boström 2018; Górska and Czesnikiewicz-Guzik 2020). The oral microbiota is key for maintaining the metabolic balance and homeostasis in the human body (Wade 2013; Sampaio-Mai et al. 2016; Bourgeois et al. 2019).

The Qinghai-Tibet Plateau is one of the most extreme environments on earth, with an average altitude of about 4000 m, and it is the highest plateau on the earth (Nikitkova et al. 2013). Hypoxia, cold, dry, high altitude, low pressure, and high ultraviolet radiation are the main characteristics of the Qinghai-Tibet Plateau (Zhang et al. 2016). This harsh living environment has caused adaptive changes in the Tibetans living on the plateau (Bigham et al. 2010; Simonson et al. 2010; Yi et al., 2010; Gnecchi-Ruscone et al. 2018). For example, Tibetans have a lower hemoglobin concentration, lower pulmonary artery pressure, lower incidence of chronic altitude sickness, higher resting lung ventilation, higher exhaled nitric oxide, and higher exercise endurance (Ge et al. 1994, 2011). Tibetans also have the *EPAS1*, *EGLN1*, and *PPARA* genes related to adaptation to the plateau, which help the body to better adapt to the harsh environment (Zhou et al. 2013; Huerta-Sánchez et al. 2014; Lou et al. 2015). Many bacteria in the Tibetan intestinal microbiota produce short-chain fatty acids, which promote energy metabolism and homeostasis of the intestinal microbiota (Jia et al. 2020).

The diversity and community structure of microorganisms are subject to selective pressures of the harsh plateau environment (Li et al. 2019). Cold, hypoxia, and high ultraviolet radiation may be important factors affecting the intestinal and skin microbiota of high-altitude populations (Li et al. 2019; Jia et al. 2020). However, only a few studies have focused on the characteristics of the oral microbiota in high-altitude populations (Leung et al. 2003), and little is known about the relationship between the high-altitude environment and the oral microbiota. Understanding the changes in the human oral microbiota along an altitude gradient will help improve human oral health at high-altitude. In this study, we compared the alpha diversity, community structure, biomarkers, gene pathways, and microbial network of the oral microbiota sampled from humans living at four altitudes (2800–4500 m above sea level).

We hypothesized that the difference in oral microbial community structure between Tibetans living at high- and ultra-high-altitudes might be caused by the selective pressure of the plateau environment. First, we tested for oral microbes that are uniquely adapted to high-altitude environments. Second, we assessed the changes in alpha and beta diversity of the oral microbiota with increasing altitude. Third, we evaluated the difference in network topology characteristics between the high- and ultra-high-altitude human oral microbial communities. Our study provides new insight into the relationship between altitude and the oral microbiota, and the characteristics of the oral microbiota in people living at ultra-high-altitude.

## Materials and methods

### Data and sample collection

A total of 167 saliva samples were collected from 167 Tibetans living at four different altitudes, including Nyingchi (2800 m), Lhasa (3650 m), Shigatse (4000 m), and Naqu and Ali (4500 m). The age, sex, height, weight, body mass index (BMI), dietary pattern (animal-based dietary pattern, plant-based pattern, animal-plant balanced dietary pattern), and ethnicity of the Tibetans were collected. All samples were collected in September 2016. The Tibetans were divided into high-altitude (< 3650 m) and ultra-high-altitude (> 4000 m) groups according to the altitude of the living environment. The sampling inclusion criteria were: (i) no oral diseases; no antibiotic use within 3 months prior to sample collection; and (iii) no food, smoking, or chewing gum within 2 h prior to sample collection. Approximately 10 mL of saliva was collected from each volunteer and stored in 50-mL sterile tubes at − 80 °C until use. All participants signed informed consent and fully understood the purpose of the study. Ethics approval was obtained by the Ethics Committee of Xizang Minzu University (ID: 201601), and written permission from all participants was obtained. The present study strictly followed the standard biosecurity and safety procedures of Xizang Minzu University.

### Bacterial DNA extraction and polymerase chain reaction (PCR)

Total microbial DNA of 167 saliva samples was extracted with the TIANamp Swab DNA Kit (Shanghai, China). The universal 341F and 805R primers were used to amplify the V3-V4 region of the 16S rRNA genes of the oral microbiota. PCR amplification was performed in triplicate in a total volume of 10 µL containing 1 µL 10× buffer, 0.8 µL dNTPs (25 mM), 0.2 µL each of the 341F and 805R primers (10 µM), 0.2 µL Toptaq DNA Polymerase, 3 µL microbial DNA, and 4.8 µL ddH2O. The PCR amplification conditions were denaturation at 94 °C for 2 min, followed by 25 cycles of denaturation at 94 °C for 30 s, annealing at 55 °C for 30 s, elongation at 72 °C for 1 min, and a final elongation at 72 °C for 10 min.

### DNA library construction and high-throughput sequencing

The PCR products were separated by 2% agarose gel electrophoresis, purified using AMPure XP beads (Beckman Coulter, Brea, CA, USA), and quantified using a Quantus™ fluorometer (Promega, Madison, WI, USA). The DNA library was obtained using a NEXTFLEX Rapid DNA-Seq Kit (Illumina, San Diego, CA, USA), merged into equimolar concentrations, and sequenced using an Illumina MiSeq platform with a 2 × 250 paired-end protocol. The raw sequence data reported in the present study were deposited in the Genome Sequence Archive at the Data Center, Beijing Institute of Genomics, Chinese Academy of Sciences, under accession number CRA003254. The shared URL is http://bigd.big.ac.cn.

### Processing of the sequencing data

The raw FASTQ files were demultiplexed, quality-filtered using Trimmomatic, and merged with FLASH according to the following criteria: (1) The reads were truncated at any site receiving an average quality score less than 20 over a 50 bp sliding window; (2) primers were exactly matched, allowing two-nucleotide mismatching, and reads containing ambiguous bases were removed; sequences with overlaps longer than 10 bp were merged according to their overlap sequence. Operational taxonomic units (OTUs) were clustered with a 97% similarity cutoff using UPARSE (version 7.1 http://drive5.com/uparse/), and chimeric sequences were identified and removed using UCHIME. The taxonomy of each 16S rRNA gene sequence was analyzed by the RDP Classifier algorithm (http://rdp.cme.msu.edu/) against the Silva (SSU123) 16S rRNA database using a confidence threshold of 80%.

### Bioinformatics and statistical analysis

R software version 3.6.2 (vegan and pheatmap software packages; The R Foundation for Statistical Computing, Vienna, Austria) was used to draw rarefaction curves and heatmap plots. The Chao 1 and ACE indices were measured using Mothur software (Schloss et al. 2009). Beta diversity was assessed by principal coordinate analysis (PCoA) and permutational multivariate analysis of variance (PERMANOVA) based on the Bray–Curtis distance (Kelly et al. 2015). A linear discriminant analysis (Segata et al. 2011) effect size (LEfSe) was carried out to highlight the biomarkers in the different groups, and the LDA score was set to 2.0. PICRUSt software (Langille et al. 2013) was employed to predict the potential functions of the oral and intestinal microbiota and analyze them in the context of the Kyoto Encyclopedia of Genes and Genomes (KEGG) database. The demographic information of the Tibetans was compared with Student’s *t*-test. The Wilcoxon test was employed to compare two groups. P-values were adjusted by the false discovery rate; q < 0.05 was considered significant. Pearson’s correlation analysis was used to calculate the correlations between the oral bacteria and altitude. The SparCC algorithm was used to estimate p-values and calculate the bacterial correlation based on the abundance of the genus in the microbial network analysis. Only correlation values with p-values < 0.05 were kept in the network. The interaction networks were visualized using Gephi 0.9.1.

## Results

### Oral microbiota composition

A total of 167 saliva samples were collected in this study. We divided the samples into the high-altitude (HA) and ultra-high-altitude (UHA) groups. No significant differences in age, height, weight, or BMI were observed between the HA and UHA groups (Table 1). The dietary structure of the UHA group was mainly characterized by the “animal based dietary pattern” (44%), and that of the HA group was also mainly characterized by the “animal based dietary pattern” (38%) (Table 1).

**Table 1:** Volunteer information

	High-altitude Tibetan	Ultra-high-altitude Tibetan
Sample size	84	83
Age (years ± SD)	19 ± 1	19 ± 1
Height	163.1 ± 7.6	163.5 ± 7.6
Weight	54.3 ± 7.9	54.5 ± 8.6
BMI	20.3 ± 2.7	20.4 ± 2.6
Gender (male/the total number of people)	11%	80%
Gender (female/the total number of people)	89%	20%
Animal based dietary pattern (proportion of the total number of people)	38%	44%
Animal-plant balanced dietary pattern (proportion of the total number of people)	37%	36%
Plant based dietary pattern (proportion of the total number of people)	25%	20%

A total of 10,567,309 sequences were obtained following quality control, with an average of 63,277 sequences (41,229−88,348) per sample, and 41,229 independent OTUs were obtained after 97% clustering. The Tibetan oral microbiota was mainly composed of *Firmicutes* (35%), *Bacteroidetes* (22%), *Proteobacteria* (21%), *Actinobacteria* (11%), *Fusobacteria* (6%), Candidatus_Saccharibacteria (1%), and *Spirochaetes* (1%). At the genus level, the oral microbiota were composed primarily of *Streptococcus* (16.24%), *Prevotella* (13.34%), *Neisseria* (9.81%), *Veillonella* (7.48%), *Haemophilus* (7.39%), *Rothia* (6.92%), *Fusobacterium* (3.64%), *Actinomyces* (2.97%), *Granulicatella* (2.47%), *Leptotrichia* (2.40%), *Gemella* (1.84%), *Porphyromona*s (1.84%), *AlloPrevotella* (1.42%), and *Atopobium* (1.02%) (mean relative abundance > 1%) (Additional file 1: Fig. S1).

### The abundance of bacteria varied with altitude

**Fig. 1:**
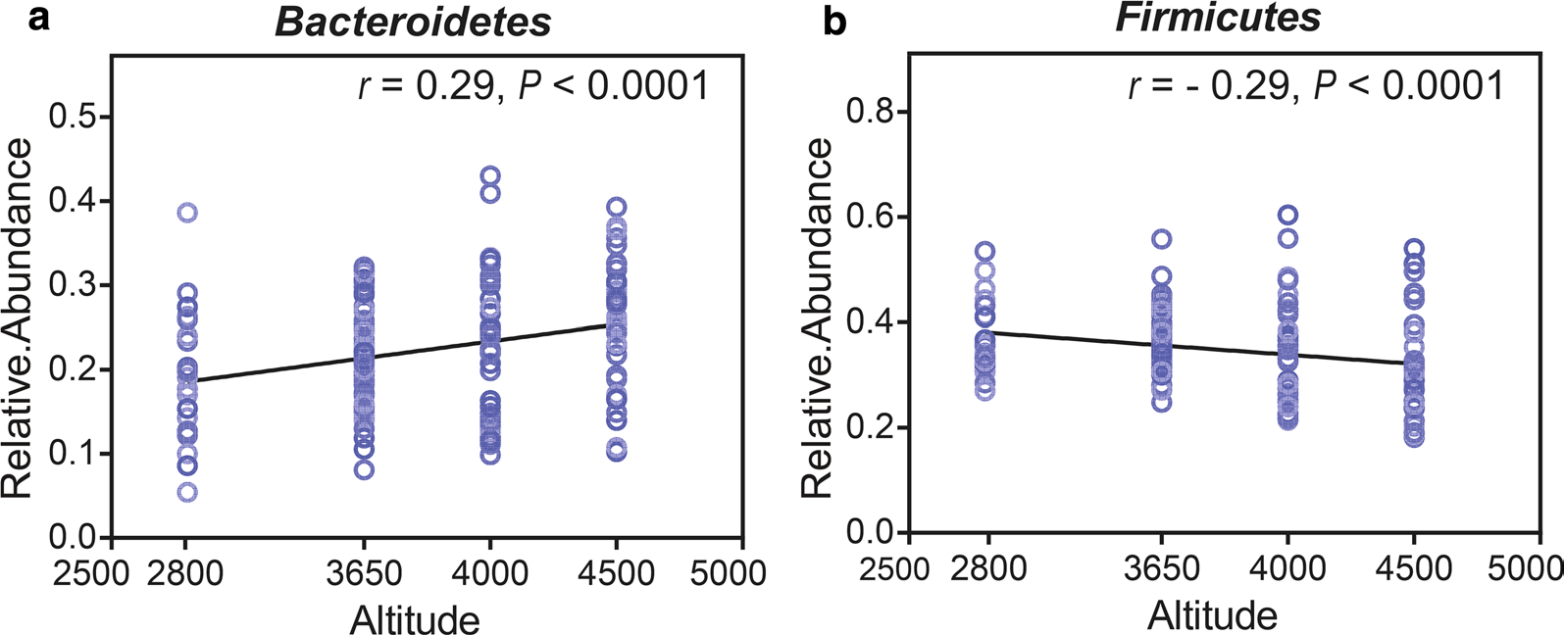
Correlation between altitude and the dominant phyla (mean relative abundance > 1%), *Bacteroidetes* (**a**) and *Firmicutes* (**b**)

**Fig. 2:**
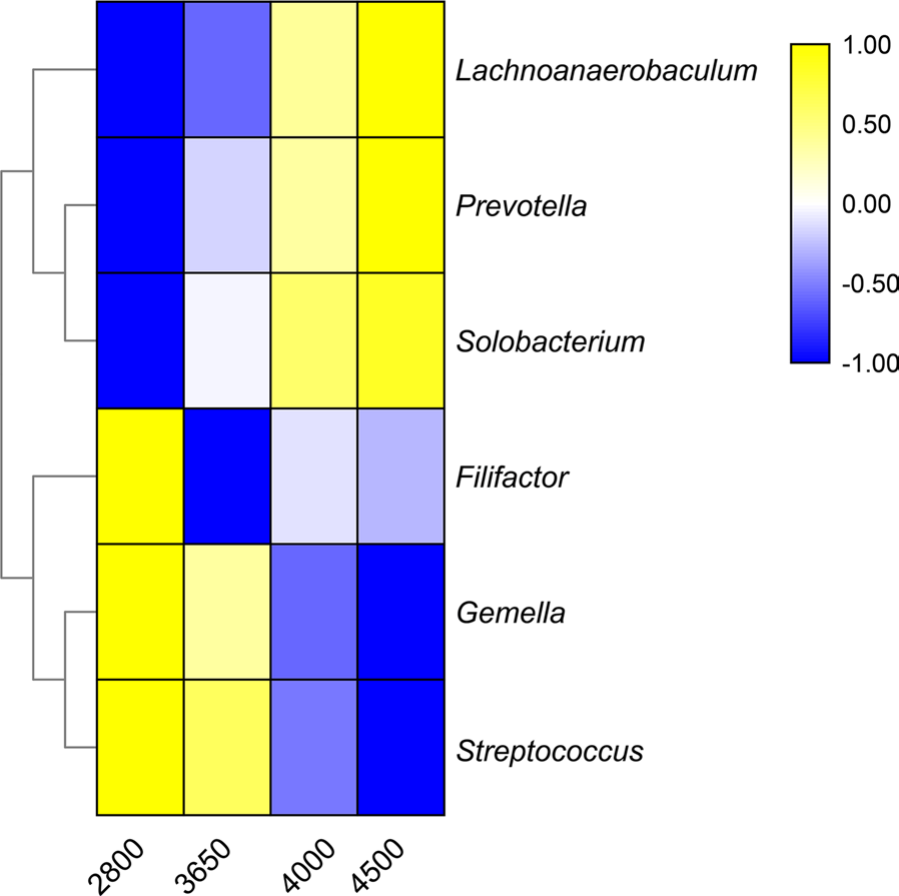
Pearson’s correlation analysis between major genera (mean relative abundance > 0.2%) and altitude

We calculated the correlation between the bacteria and altitude. As results, the abundance of *Bacteroidetes* (r = 0.29, P < 0.0001) increased and the abundance of *Firmicutes* (r =  − 0.29, P < 0.0001) decreased with altitude (mean relative abundance > 1%, Fig. 1a, b). The relative abundance of *Prevotella*, *Solobacterium*, and *Lachnoanaerobaculum* increased with altitude (all r > 0.22, P < 0.005). In contrast, the relative abundance of *Streptococcus*, *Gemella*, and *Filifactor* decreased with altitude (all mean relative abundance > 0.2%, all r <  − 0.26, P < 0.001, Fig. 2).

**Fig. 3:**
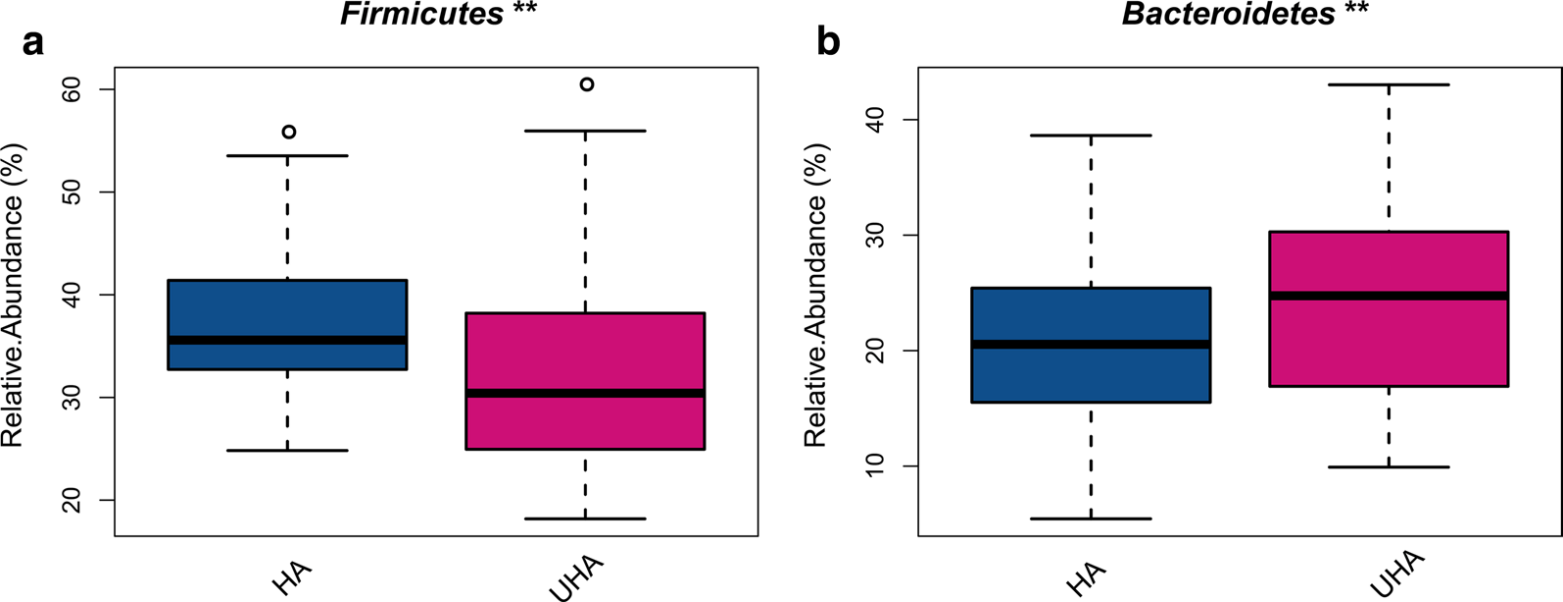
Differences in relative abundance of bacterial phyla between the high altitude (HA) and ultrahigh altitude (UHA) groups

**Fig. 4:**
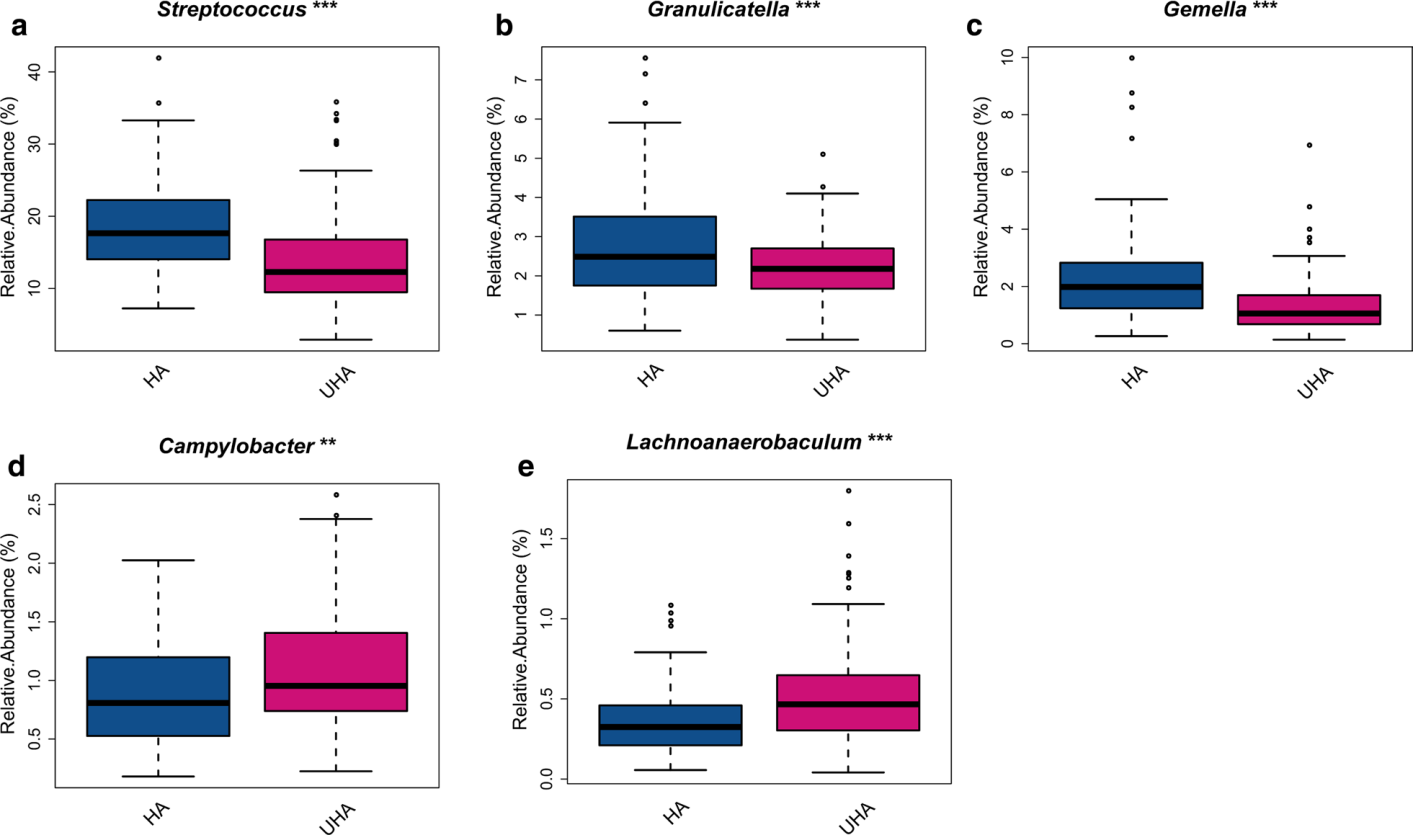
Differences in relative abundance of bacterial genera between the high altitude (HA) and ultrahigh altitude (UHA) groups

The predominant bacteria were largely inconsistent between the HA and UHA groups. The relative abundance of *Firmicutes* decreased in the UHA group, whereas the relative abundance of *Bacteroidetes* increased (relative abundance > 20%, q < 0.0001, Fig. 3a, b). The relative abundance levels of *Streptococcus*, *Granulicatella*, and *Gemella* were higher in the HA group (relative abundance > 1%, q < 0.05). In contrast, the relative abundance levels of *Lachnoanaerobaculum* and *Campylobacter* group were higher in the UHA group (relative abundance > 0.8%, q < 0.05, Fig. 4a–e).

**Fig. 5:**
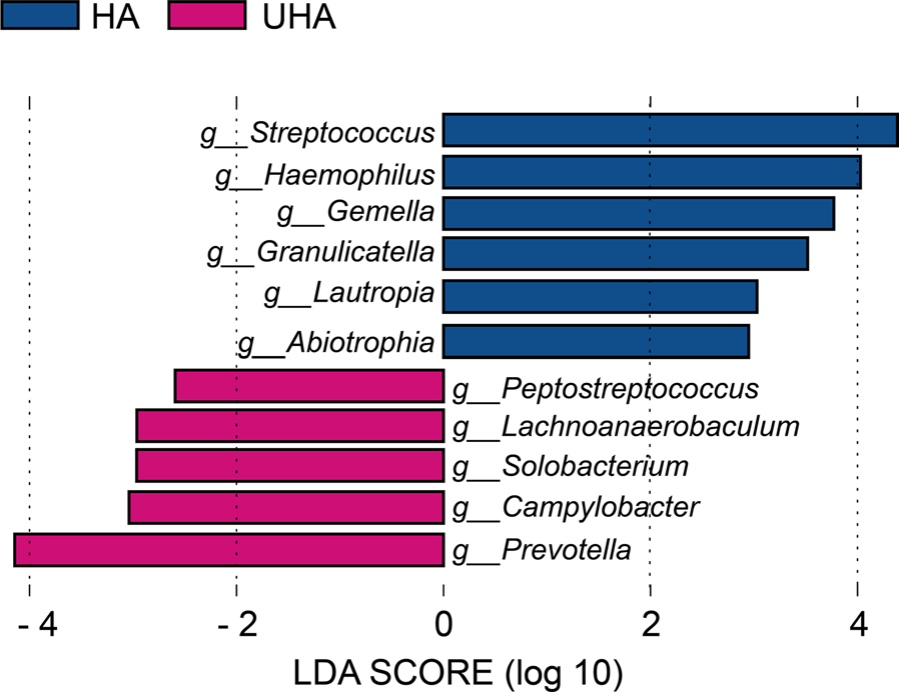
LEfSe was used to compare the abundance of bacterial genera between the groups

The LEfSe analysis demonstrated that the taxa were significantly different between the HA and UHA groups. The histograms in Fig. 5 represent potential biomarkers of the different groups. At the genus level, *g_Abiotrophia, g_Gemella, g_Granulicatella, g_Haemophilus, g_Lautropia, g_Streptococcus* were significantly enriched in the HA group, whereas *g_Campylobacter, g_Lachnoanaerobaculum, g_PeptoStreptococcus, g_Prevotella, g_Solobacterium* were significantly enriched in the UHA group (LDA > 2, P < 0.05).

### Variations in oral microbiota diversity with altitude

**Fig. 6:**
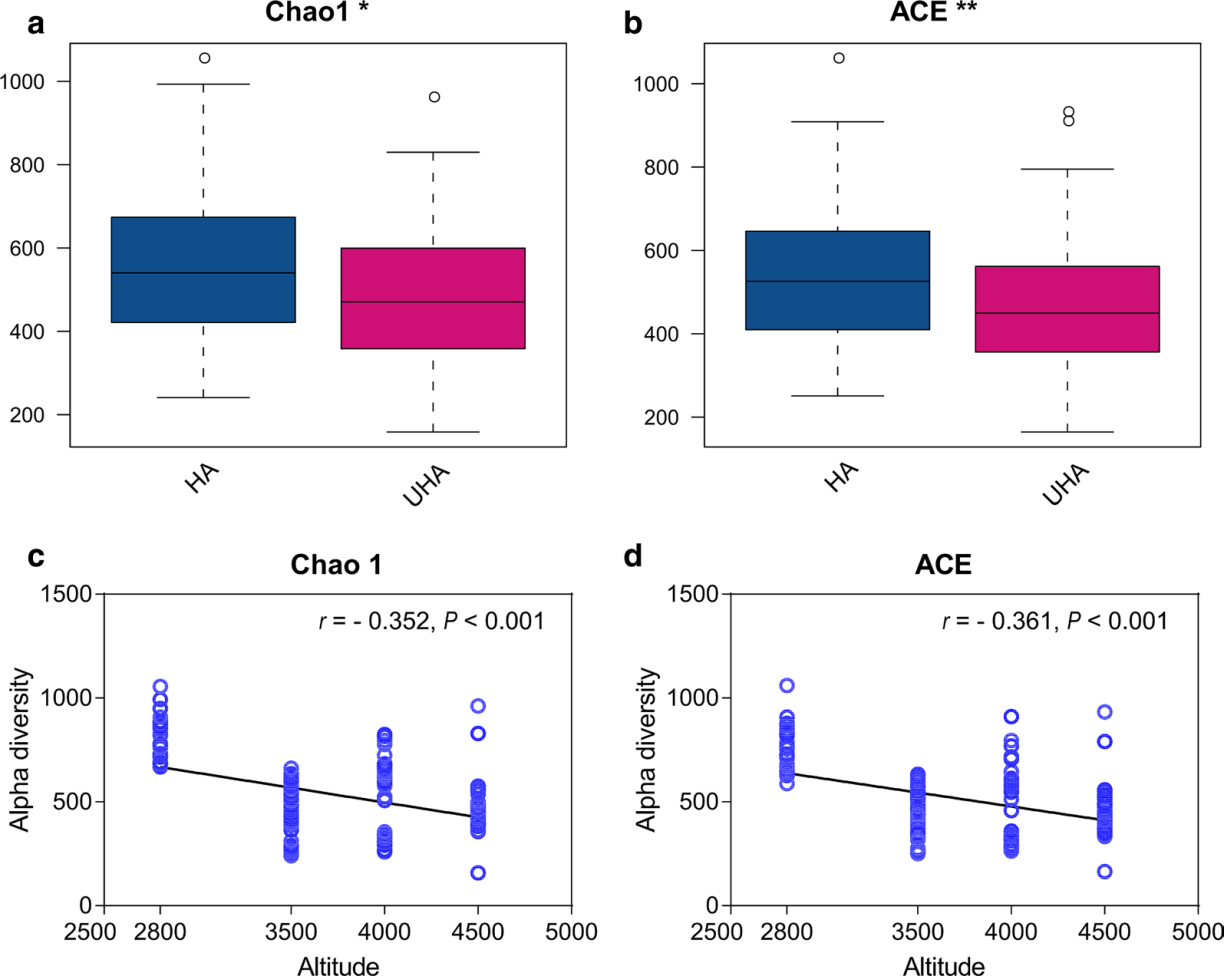
Comparison of alpha diversity between the high altitude (HA) and ultrahigh altitude (UHA) groups (**a**, **b**). Boxplots showing the differences in the alpha diversity indices (Chao 1 and ACE) between the high altitude (HA) and ultrahigh altitude (UHA) groups. Pearson’s correlation analysis between altitude and the alpha diversity indices Chao 1 (**c**) and ACE (**d**)

Additional file 1: Figure S2 shows that the rarefaction curves of all samples tended towards saturation. Alpha diversity indices, such as the Chao1 and ACE, are used to assess the diversity of bacteria within a community. The Chao1 and ACE indices of the oral microbiota were significantly different between the HA and UHA groups. The alpha diversity of the HA group was higher than that of the UHA group (Fig. 6a, b). In addition, altitude was negatively correlated with ACE (r =  − 0.352, P < 0.001) and Chao1 (r =  − 0.361, P < 0.001) of the Tibetan oral microbiota (Fig. 6c, d). In other words, alpha diversity decreased with altitude.

**Fig. 7:**
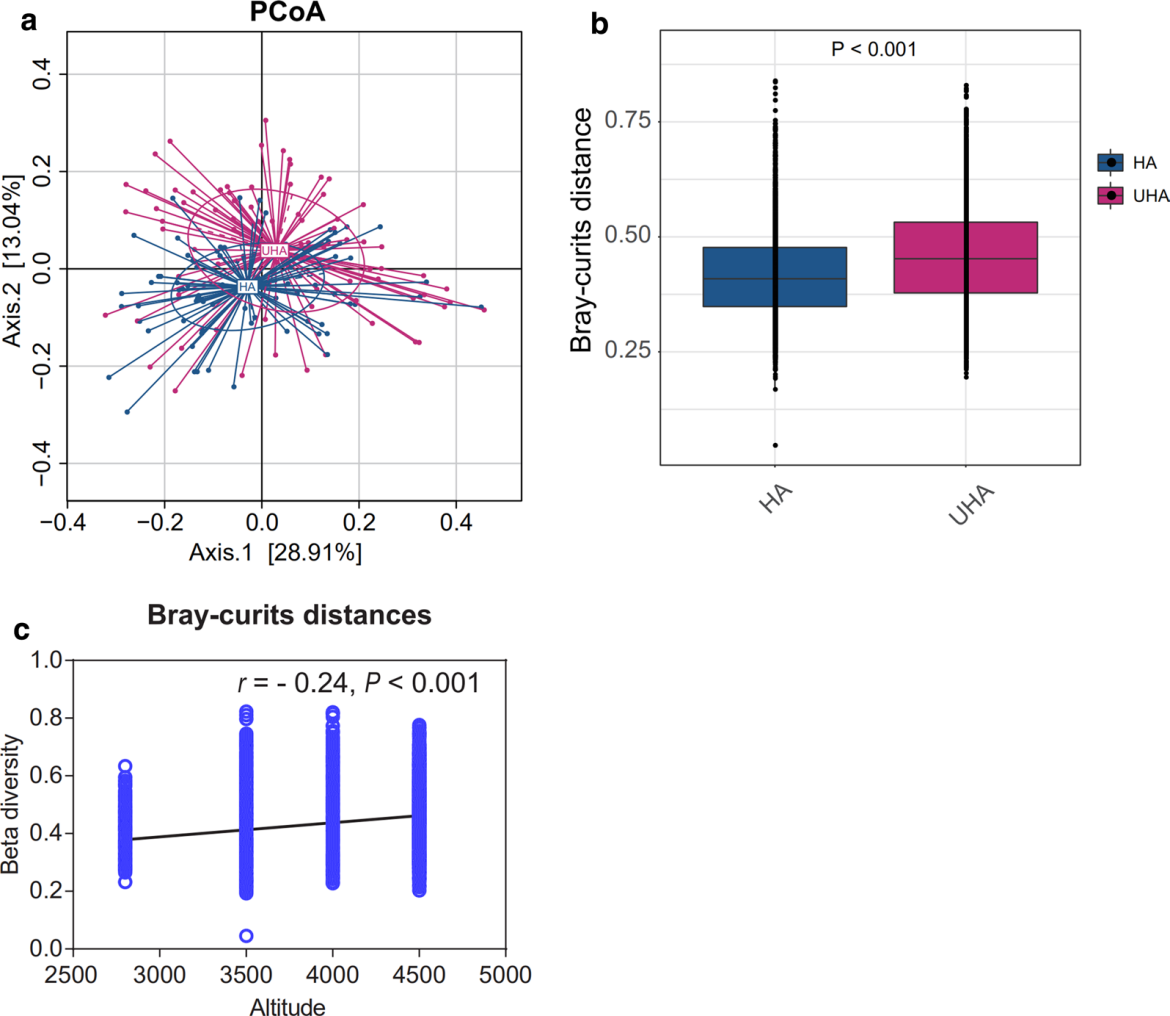
The relationship between beta diversity and altitude. Beta diversity was accessed using a **a** principal component analysis plot and **b** PERMANOVA based Bray–Curtis distances. **c** Pearson’s correlation analysis between altitude and beta diversity (Bray–Curtis distances)

A beta diversity analysis was conducted to assess the composition of the microbial communities between samples from the two high altitude groups. The PCoA plot and PERMANOVA analysis showed that the oral microbiota structure was significantly different (R^2^ = 0.031, P < 0.001), and a low clustering similarity was detected between the HA and UHA groups (Fig. 7a, b). Altitude was positively correlated with beta diversity (r = 0.24, P < 0.001) of the Tibetan oral microbiota (Fig. 7c). We demonstrated that beta diversity increased with altitude (Bray–Curtis distance).

### Differences in gene function between the high- and ultra-high-altitude oral microbiota

**Fig. 8:**
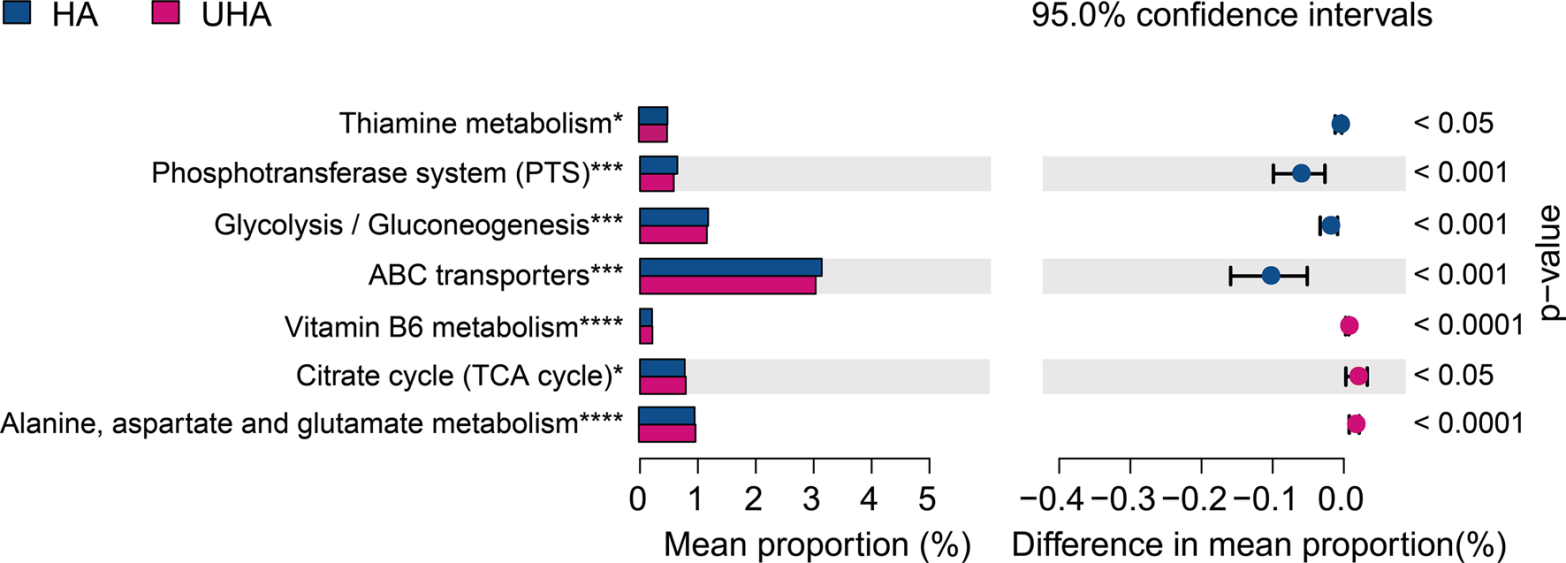
Comparison of predicted gene functions of oral microbiota between the high altitude (HA) and ultrahigh altitude (UHA) groups

A PICRUSt analysis was performed to evaluate the effect of altitude on oral microbiota function. Samples from the HA and UHA groups had different KEGG profiles, indicating dissimilar microbial functional features. The alanine, aspartate, and glutamate metabolic, as well as the vitamin B6 metabolic pathways were upregulated in the UHA group. In contrast, gene functional pathways involved in thiamine metabolism, the phosphotransferase system, glycolysis/ gluconeogenesis, and ABC transporters were enriched in the HA group (Fig. 8).

### Differences in the microbial network between high- and ultra-high-altitude Tibetans

**Fig. 9:**
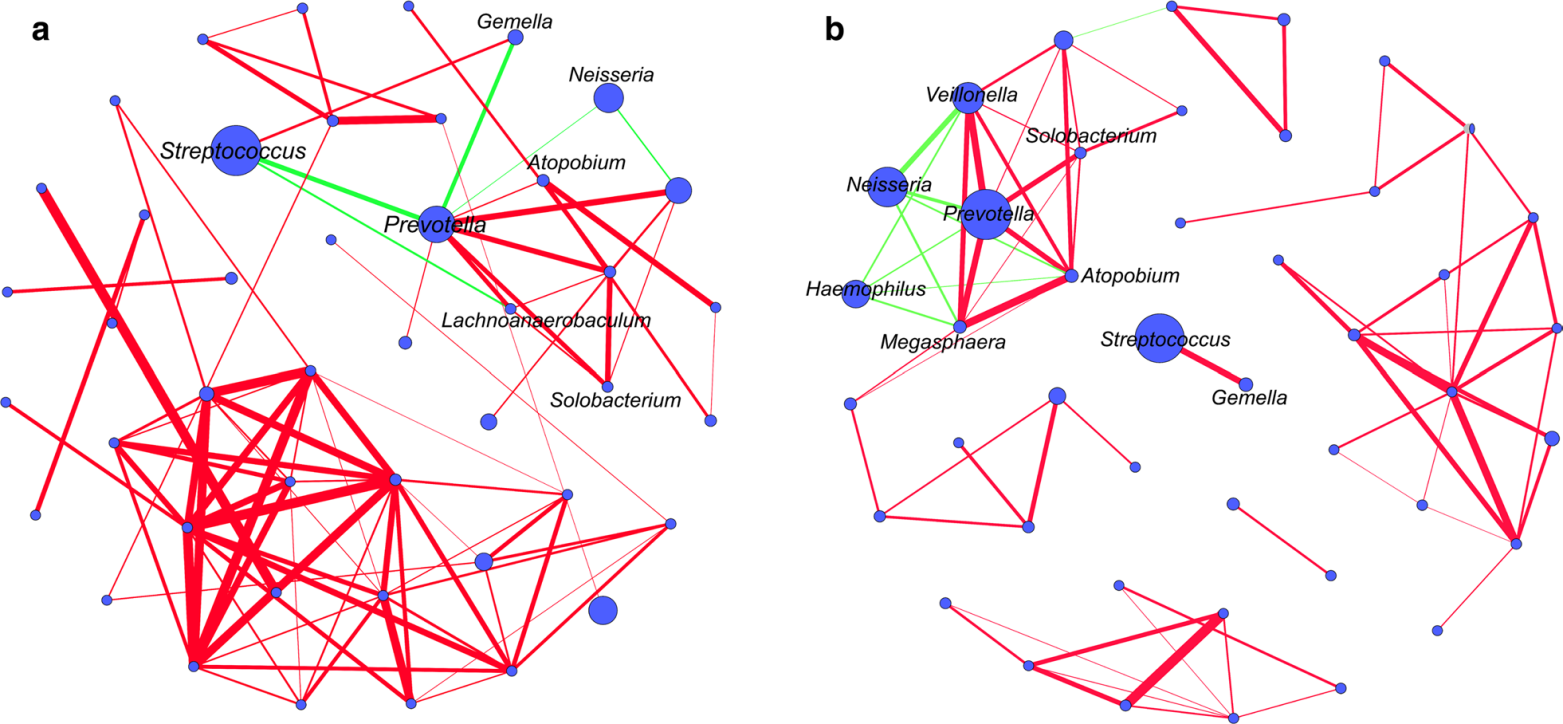
Different interactions between bacterial genera were compared in the microbial networks of the high altitude (HA) and ultrahigh altitude (UHA) groups. **a** High-altitude Tibetans. **b** Ultra-high-altitude Tibetans. Nodes represent bacterial genera, and the size represents the relative abundance (larger circle indicates greater abundance). Red line: positive correlation; blue line: negative correlation. Relative thickness of the lines represents the degree of correlation (greater thickness of the edges means a stronger correlation)

Analyzing the microbial network helped to understand the interaction between bacterial groups. The composition and topography of the UHA microbial network were quite distinct from those of the HA microbial network. The most significant difference originated from the *Streptococcus* and *Prevotella* associations with other genera in the microbial network. Our study determined that *Solobacterium* was positively correlated with *Prevotella*. *Gemella* was positively correlated with *Streptococcus*, but negatively correlated with *Prevotella*. We computed the number of edges and nodes between the microbial networks of the HA and UHA groups. The HA oral bacterial network (63 nodes and 90 edges) had more nodes and edges than those of the UHA network (61 nodes and 76 edges). The most significant difference was in the association between *Streptococcus* and *Prevotella* and other bacteria in the microbial network. These results indicate that the composition and topography of the oral microbial network was affected by altitude (Fig. 9a, b).

## Discussion

The oral cavity communicates with the external environment (Diamond et al. 2008; Aleti et al. 2019), so the external environment is one of the most important contributors affecting the oral microbiota (Gilbert and Stephens 2018; Willis et al. 2018). The extreme environment of the Tibetan Plateau has exerted tremendous evolutionary pressure on the human microbiota, thereby affecting microbial diversity and community composition. The oral microbiota plays a key role maintaining the human health (Grant et al. 2010; Gomez and Nelson 2016; Sampaio-Mai et al. 2016; Lamont et al. 2018; Bourgeois et al. 2019). Many metabolic diseases can be associated with an imbalance in the oral microbiome (Xiao et al. 2017; Wang et al. 2018; Lv et al. 2020). Thus, it is necessary to explore the relationship between the oral microbiota and human health. Our study was performed to reveal: (1) differences in alpha and beta diversity of the oral microbiota between the HA and UHA groups; (2) differences in the abundance of genera, biomarkers, the microbial network, and gene pathways between the HA and UHA groups; and the effect of increasing altitude on the abundance and diversity of bacterial genera. The majority of the population living on the plains are Han, while Tibetans mainly live in the Qinghai-Tibet Plateau. The host oral microbiota is influenced by genetic background. We did not recruit Han from the plains to avoid interference of the host genetic background in this study.

### Comparison of alpha and beta diversity of the oral microbiota in high- and ultra-high-altitude Tibetans

Species diversity is positively correlated with ecosystem stability. Therefore, an ecosystem will have a higher level of ecosystem functions and services. Previous studies have shown that higher species diversity reduces the possibility of loss or extinction of species because the higher species diversity buffers ecosystem functions (Bannar-Martin et al. 2018; Louca et al. 2018).

Our study showed that alpha diversity of the oral microbiota decreased with altitude, indicating that a higher prevalence of oral diseases might occur in Tibetans at high altitudes (Guan et al. 2020; Hou et al. 2014; Wu et al. 2020). Some studies have demonstrated that the oral microbiota is consistent with the intestinal microbiota, possibly because most oral bacteria can colonize the intestine (Atarashi et al. 2017; Cao 2017; Schmidt et al. 2019; Kitamoto et al. 2020). The alpha diversity of the skin and intestinal microbiota in high-altitude humans and animals is significantly lower than that of low-altitude humans and animals due to diet and environmental factors (Li et al. 2019). In this study, no significant differences in age, height, weight, BMI, or eating habits were observed between the HA and UHA groups. Therefore, we speculated that the difference in diversity between the HA and UHA groups might be caused by altitude factors.

A low-oxygen environment increases the work load on the human heart; thus, native Tibetans have a higher prevalence of heart disease. Chronic intermittent hypoxia (CIH) damages the heart function of guinea-pigs and alters the intestinal microbiota. The alpha diversity of the intestinal microbiota of CIH mice is significantly lower than that of normal mice (Lucking et al. 2018). Moreover, exposing the host to CIH could induce oxidative stress and the inflammatory response, which contribute to changes in composition of the microbiota and decrease alpha diversity (Wu et al. 2016; Lucking et al. 2018). Our results support this view that alpha diversity of the microbiota decreased with oxygen concentration. The colonization of oral bacteria was compromised by the concentration of oxygen, suggesting that oral microbial colonization is related to oxygen concentration. We speculate that anaerobic bacteria dominate the microbial community in the hypoxic high-altitude environment. The colonization of aerobic bacteria in the oral cavity was inhibited by hypoxia, resulting in decreased alpha diversity of the oral microbiota.

The alpha diversity of a microbial community can decrease with temperature (Chevalier et al. 2015; Moreno-Navarrete and Fernandez-Real 2019). The alpha diversity of the oral microbiota in an Alaskan population living in a cold environment is significantly lower than that of a German population living at a higher temperature (Jing et al 2014). The oral microbiota is affected by the environmental temperature through respiration, and temperature is a critical factor affecting bacterial abundance. The optimal temperature for growth of most parasitic microorganisms on the human body is around 37 °C (Grice et al. 2008), and low-temperature will decrease the rate of bacterial growth. We speculate that the decreased alpha diversity of the oral microbiota may have been caused by the cold air in the ultra-high-altitude environment.

The oral cavity is region where air is exchanged between the outside world and the human body (Diamond et al. 2008; Aleti et al. 2019), suggesting that the oral microbiota is much more susceptible to environmental exposure than any other human microbiota. Previous studies have documented similar findings that the alpha diversity of microbiota is notably correlated with latitude (Nasidze et al. 2009). The bacteria in the plateau environment must withstand the influence of high ultraviolet radiation, hypoxia, cold, and other factors, and the species and numbers of oral microbiota obtained from the living environment also decrease (Li et al. 2019). Thus, we speculate that the difference in diversity between the HA and UHA groups may have been caused by altitude factors. The alpha diversity of the UHA group was significantly lower than that of the HA group, which might be due to the difference in the living environment of the microbiota.

Our results show that oral microbial beta diversity increased with altitude, indicating that the community dissimilarity between the HA and UHA groups was more distinct with increasing altitude. Previous research has indicated that hypoxia initiates inflammation and an immune response, and produces amylase, protease, immunoglobulin and other metabolites, which could indirectly change beta diversity (Hanski et al. 2012; Adak et al. 2013). Similarly, other studies have reported a positive correlation between skin microbiota beta diversity and altitude, and that beta diversity might be affected by altitude (Li et al. 2019).

High beta diversity represents sample heterogeneity, which, in turn, may be the result of the size of the sample collection area. In our study, the UHA group originated from a far larger geographic area, while the HA group was relatively concentrated, and individuals were relatively close together. Moreover, previous studies have reported that the beta diversity of samples from large areas is higher than that of samples from small areas, which might be due to the higher environmental heterogeneity of large areas relative to small areas (Jing et al. 2014). To sum up, hypoxia, altitude, and geographical area were the essential factors affecting beta diversity. Our study shows that alpha and beta diversity are vulnerable to altitude.

### Comparison of the abundance of bacterial genera between high and ultra-high-altitude Tibetans

In this study, the abundance of *Firmicutes* decreased with altitude, while the abundance of *Bacteroidetes* increased with altitude, which agreed with previous studies. The maximal oxygen consumption (VO_2_max) of the human body decreases with altitude (Squires et al. 1982). Durk et al. (2019) showed that a lower *Firmicutes*/*Bacteroidetes* ratio is related to a lower VO_2_max. The blood pressure of humans’ increases with altitude, and microorganisms are involved in the regulation of blood pressure (Li et al. 2017; Stoltzfus et al. 2020; Yang et al. 2015). The lower abundance of *Fimicutes* and the higher abundance of *Bacteroidetes* help the host maintain normal blood pressure (Lucking et al. 2018). Recent studies have suggested an association between changes in the oral and intestinal microbiota and low temperature and hypoxia (Bhushan et al. 2019; Sommer et al. 2016; Zhang et al. 2018). Previously, it was shown that cold and hypoxia might result in a changed abundance of *Firmicutes* and *Bacteroidetes* (Bhushan et al. 2019; Sommer et al. 2016; Zhang et al. 2018). We speculate that the abundances of *Firmicutes* and *Bacteroidetes* decrease and increase significantly with altitude, respectively, due to the VO_2_max, blood pressure, cold, and hypoxia.

Our data show that the abundance of *Prevotella* was higher in the UHA group, while the abundance of *Streptococcus* was lower, which was confirmed by earlier studies. Short chain fatty acids are promoted by *Prevotella*, which reduce blood pressure and pulmonary hypertension (Li and Zhao 2015). Zeng et al. (2020) revealed that *Prevotella* help humans to better adapt to the extreme high altitude environments. *Prevotella* induce significant immune responses and stimulate the production of cytokines by oral epithelial cells. *Prevotella* increases the immune response to produce more cytokines (IL-8 and TNF-α) under hypoxic conditions (2% oxygen level). In contrast, *Prevotella* decreases the immune response and the production of cytokines is lowest at a 21% oxygen concentration (Grant et al. 2010). Humans often feel a dry mouth and tongue at high altitude, because high altitude air is drier than low altitude air. Similar symptoms have been reported by other studies, e.g., Sjogren’s syndrome patients also often have a dry mouth. The abundance of *Prevotella* is significantly higher in Sjogren’s syndrome patients than in healthy subjects (Rusthen et al. 2019). The temperature of the high-altitude environment is higher than that of the ultra-high-altitude environment. Temperature is an indispensable driving force shaping the abundance of *Streptococcus*. The optimal temperature for growth of *Streptococcus* is about 37 °C. Previous studies have found that *Streptococcus* is highly abundant in the intestines of animals and humans at low altitudes (Zeng et al. 2020). Thus, we speculate that the abundance of *Prevotella* and *Streptococcus* was affected by dry air, hypoxia, cold, and other environmental factors.

Our data show that the abundances of *Lachnoanaerobaculum* and *Solobacterium* increased, whereas the abundance of *Filifactor* decreased with altitude. *Lachnoanaerobaculum* and *Solobacterium* are associated with periodontitis (Nowicki et al. 2018; Shaddox et al. 2012). We speculate that the high-altitude environment caused insufficient blood perfusion in the periodontal tissues, which affected the integrity of the periodontium, leading to increased abundance of *Lachnoanaerobaculum* and *Solobacterium*, which are pro-inflammatory genera (Karl et al. 2018; Xiao et al. 2012). *Filifactor* encodes superoxide reductase to scavenge superoxide radicals, and protect itself from oxidative stress (Mishra et al. 2020a).

### Comparison of bacterial gene functions between high- and ultra-high-altitude Tibetans

PICRUSt provides insight into the effects of altitude on the functions of oral bacterial communities. Notably, the vitamin B6 metabolic pathway was enriched in the UHA group, and the thiamine metabolic pathway was enriched in the HA group. A high level of vitamin B6 could help the human body eliminate reactive oxygen species, avoid damage from oxidative stress, and adapt to the harsh external environment (Hellmann and Mooney 2010). Other studies have demonstrated that the vitamin B6 pathway in the skin microbiota of high-altitude people is upregulated (Li et al. 2019). Thiamine is a key player in cell metabolism (Agus et al. 2018; Spencer et al. 2019), which is related to the tricarboxylic acid cycle and glycolysis. Thiamine regulates the production of acidic substances in the oral cavity, and is a critical factor for maintaining oral health (Roager and Licht 2018). Thus, we speculate that vitamin B6 could help Tibetans adapt to the ultra-high-altitude environment.

### Changes in the network topological features with altitude

A network analysis provides a meaningful framework for revealing the complex interactions among oral bacterial genera. The network is also the foundation of alpha and beta diversity stability. Edges and nodes are the key factors when constructing microbial networks. The greater the number of edges and nodes, the more tightly and stably the microbial network structure is constructed (Faust and Raes 2012). *Prevotella* was used as the main node to build the microbial network of the UHA group. *Streptococcus* was a dominant member of the HA microbial network. We observed an interesting opposing relationship between *Prevotella* and *Streptococcus*. The abundance of *Prevotella* was inhibited by H_2_O_2_ produced by *Streptococcus*. Moreover, *Streptococcus* and *Prevotella* are inhibited even more under aerobic conditions (Yamada et al. 2010; Herrero et al. 2016; Hernandez-Sanabria et al. 2017). Networks with more edges and nodes in healthy subjects are more stable than networks in subjects with dental caries.

Our study determined that *Solobacterium* was positively correlated with *Prevotella*. The highly active β-galactosidase in *Solobacterium* provides nutrients for *Prevotella*, and promotes biofilm production (Barrak et al. 2020). *Solobacterium* and *Prevotella* produce H_2_S, which regulates human blood pressure and pulmonary arterial pressure, increases cerebral blood flow, and helps the host adapt to a low-pressure hypoxic environment (Chen et al. 2020; Mishra et al. 2020b; Prabhakhar and Joyner 2014). The present study found that *Gemella* was positively correlated with *Streptococcus*, but negatively correlated with *Prevotella*. Previous studies have reported that H_2_S is a metabolite of *Prevotella* and is positively correlated with the abundance of *Prevotella* (Basic et al. 2015; Ye et al. 2019). Yang et al. clearly demonstrated that H_2_S is negatively correlated with the abundance of *Gemella* and *Streptococcus* (Willis et al. 2018; Yang et al. 2013). We speculate that *Prevotella*, *Gemella*, and *Streptococcus* interactions are modulated by H_2_S.

Similarly, Li et al. (2019) reported that the microbial networks with more clustering coefficients in low altitude human skin are more stable than high-altitude human skin. Our results show that the microbial network structure was more compact and complex, and the interaction between the bacterial genera was more intense in the HA group than in the UHA group. Accordingly, we speculate that hypoxia, cold, and high altitude might lead to a more fragile oral microbial network. Thus, the ultra-high-altitude Tibetans were more likely to suffer from oral diseases.

In conclusion, the results of our study show that microbial composition, diversity, community structure, function, and network of the oral microbiota were affected by altitude. However, our study was only preliminary. A larger sample size is required to confirm our preliminary conclusions. Furthermore, future work should explore the relationship among altitude, oral microbiota, and oral health.

## Supplementary Information

**Additional file 1: Fig. S1.** The oral microbiota composition of the Tibetans at (A) phylum and (B) genus levels.** Fig. S2.** Rarefaction curves of the 167 Tibetan oral samples.

## Data Availability

The raw sequence data reported in the present paper have been deposited in the Genome Sequence Archive at the Data Center, Beijing Institute of Genomics (BIG), Chinese Academy of Sciences, under accession numbers CRA003254. The shared URL is http://bigd.big.ac.cn.

## Abbreviations

HA: High-altitude
UHA: Ultra-high-altitude
BMI: Body mass index
OTU: Operational taxonomic unit
PCoA: Principal coordinates analysis
PERMANOVA: Permutational multivariate analysis of variance
LEfSe: Linear discriminant analysis effect size
KEGG: Kyoto Encyclopedia of Genes and Genomes
