# Supplementary material for: Effects of altitude on human oral microbes
Source: AMB Express. 2021 Mar 6;11:41. doi: 10.1186/s13568-021-01200-0 (PMC7936934; doi:10.1186/s13568-021-01200-0)
Supplement: Supplementary file 1 — Additional file 1: Fig. S1. The oral microbiota composition of the Tibetans at (A) phylum and (B) genus levels. Fig. S2. Rarefaction curves of the 167 Tibetan oral samples. [file 13568_2021_1200_MOESM1_ESM.doc]

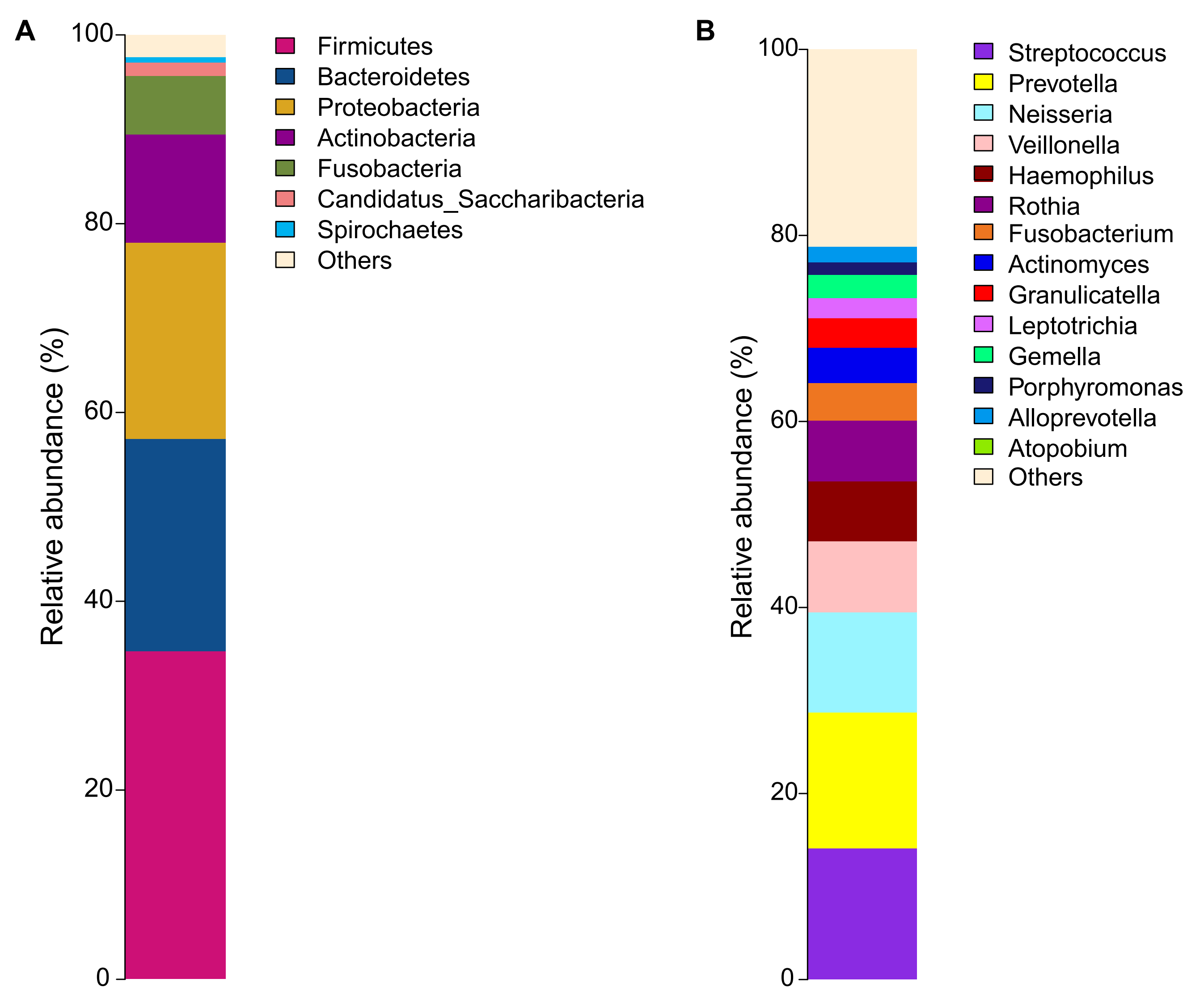


**Supplementary Fig. S1** The oral microbiota composition of the Tibetans at (A) phylum and (B) genus levels.


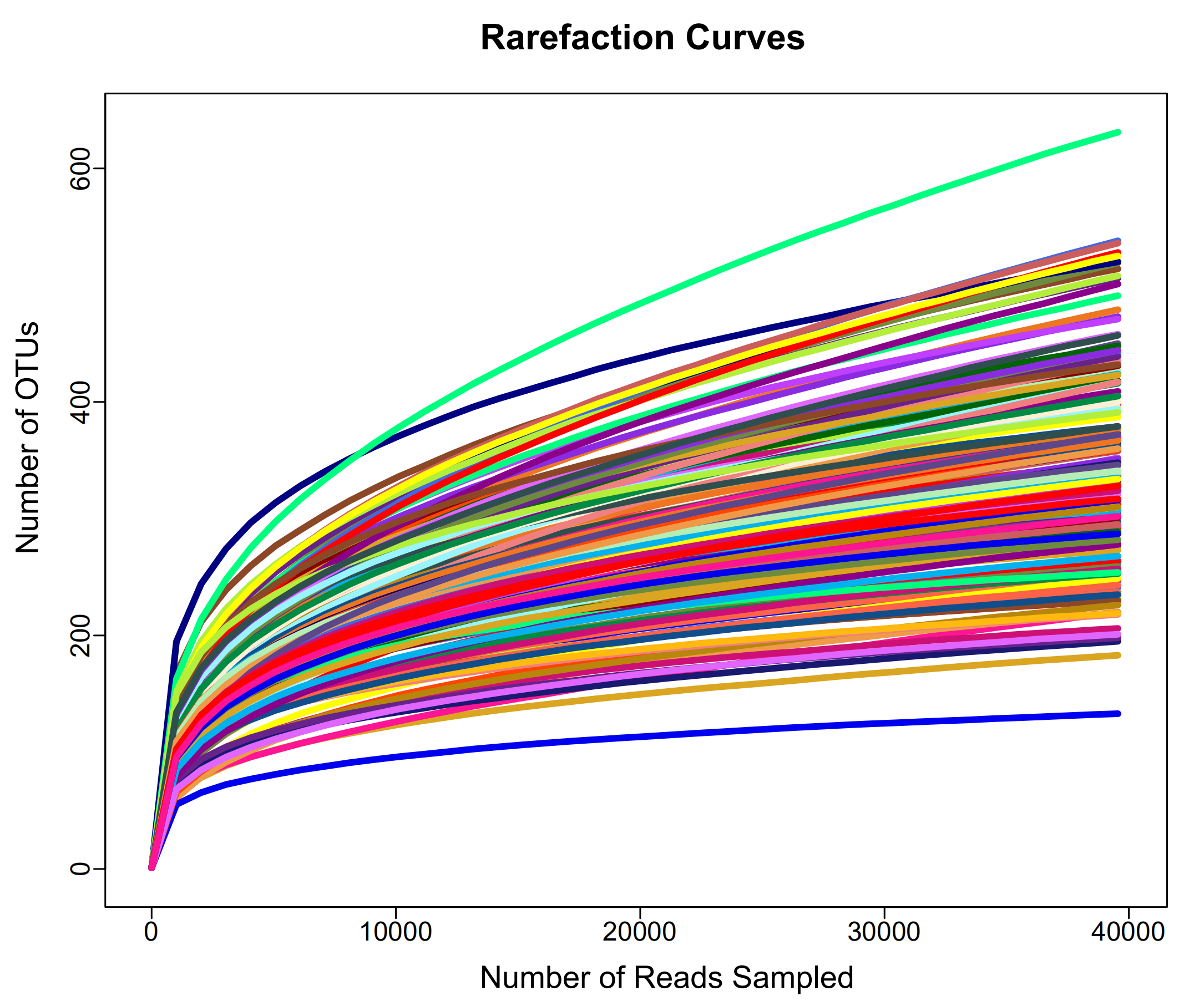


**Supplementary Fig. S2** Rarefaction curves of the 167 Tibetan oral samples.
